# Case report: Boundaries of oncological and traumatological medical care in ancient Egypt: new palaeopathological insights from two human skulls

**DOI:** 10.3389/fmed.2024.1371645

**Published:** 2024-05-29

**Authors:** Tatiana Tondini, Albert Isidro, Edgard Camarós

**Affiliations:** ^1^Institute for Archaeological Sciences, Eberhard Karls University of Tübingen, Tübingen, Germany; ^2^Department of Archaeology, University of Cambridge, Cambridge, United Kingdom; ^3^Hospital Universitari Sagrat Cor, Barcelona, Spain; ^4^Departament de Cirurgia i Especialitats Medicoquirúrgiques, Facultat de Medicina, Universitat de Barcelona, Barcelona, Spain; ^5^Department of History (Prehistory Unit), Universidade de Santiago de Compostela, Santiago de Compostela, Spain; ^6^Interuniversity Research Centre for Atlantic Cultural Landscapes (CISPAC), Santiago de Compostela, Spain

**Keywords:** ancient Egypt, neoplasms, malignancy, tumours, metastasis, cranial trauma, history of medicine, palaeopathology

## Abstract

The present case studies report malignant neoplastic and traumatic lesions observed on two ancient Egyptian skulls held at the Duckworth Collection (Cambridge University). The analysis aims to characterise the lesions and provide a diagnosis using a methodology based on micro-CT scanning and microscopic bone surface analysis. Results pointed towards neoplastic lesions in both cases and healed severe skull trauma in one of them suggesting successful traumatological therapy. Interestingly, our analysis has identified the presence of perimortem cutmarks associated with metastatic lytic lesions in one of the skulls, indicating a potential surgical treatment attempt or postmortem medical exploration. We argue that the two cases, although not contemporary, allow a palaeopathological discussion on oncological and traumatological understanding and management of such conditions in the past. The confrontation of two potential managements represented by two different types of lesions represent a clear boundary in ancient Egyptian medical care and a milestone in the history of medicine.

## 1 Introduction

Egypt is well-known for its medical knowledge and treatment modalities from both bioarchaeological [e.g., (1)] and historical written sources [e.g., The Edwin Smith Papyrus (1,700–1,600 BCE); Kahun papyri (1,850–1,700 BCE) or Ebers papyri amongst others. See (2) for an updated review]. Therefore, this is an exceptional historical context to explore the boundaries related to medical treatise and care. As an example, it is clear according to preserved papyri and hieroglyphs that ancient Egyptian medicine was advanced enough to describe, classify and successfully treat specific diseases and traumatic injuries, including bone trauma [e.g., (3–7) amongst others]. Additionally, the study of human remains from ancient Egyptian civilization offers a unique lens through which to explore the evolution of medical and healthcare practises in the past (5, 8), as it has been recognised as one of the oldest practises documented (9). Ancient palaeopathological evidence of such advancements can be seen in cases of trepanations [e.g., (10)], protheses [e.g., (11)], dental filling [e.g., (12)] and healed fractures [e.g., (13)] as examples of potential therapies and surgeries also described in the historical sources. However, the medical recognition in ancient Egyptian medicine of what we nowadays call malignancy is uncertain [(5): 81], despite the description and mention of tumours, swellings, “eating” lesions and potential matching diagnosis and treatments [see (5, 9)]. Thus, ancient Egyptian Medicine cultivated one of the most advanced medical knowledge bases in Antiquity, and still cancer represented a clear medical frontier concerning diagnosis and treatment.

Cancer is defined in modern medicine as a genetic disease comprising a wide range of conditions wherein cells begin to uncontrollably proliferate throughout the body (*National Cancer Institute*, USA). Hence, it constitutes a complex reality rather than a singular disease (14), complicating its recognition and management until very recent times. However, as previously mentioned, neoplasms were identified to some extent in the past [see (9)]. Moreover, malignancy is recognised in the ancient Egyptian palaeopathological record [e.g., (2, 15–18)], contributing to the current perspective that cancer was much more prevalent than previously assumed [see (14, 19, 20)]. In this context, considering the anatomical and physiological knowledge attained by the ancient Egyptians through medical and mummification practises, it seems reasonable to infer that some form of exploration and therapeutic attempts related to malignancy might have been developed, suggesting potential surgical management (9).

Here, we report two cases of ancient Egyptian skulls from different dynasties that allow tracing the boundaries of medical knowledge and treatment in the past. Both human remains are held at the Duckworth Laboratory (University of Cambridge, UK): Skull E270 (Late Period, 664–343 BCE) evidences a primary neoplasm and several healed cranial fractures, these last lesions showcasing the capacity of ancient Egyptian medicine to manage severe skull trauma; and Skull 236 (Old Kingdom, 2,687–2,345 BCE) reveals a primary and secondary neoplasms, actually one of the oldest known cases of malignancy from ancient Egypt, previously analysed by Calvin Wells (21). Our analysis also revealed perimortem cutmarks associated with several metastatic lesions. We argue that such modifications may be related with a perimortem surgical treatment attempt or a postmortem medical exploration, raising critical questions about the early understanding and management of oncological disorders in the history of medicine. The present research aims to approach the frontiers of ancient medicine concerning oncological and traumatological care through the palaeopathological analysis of these two cases.

## 2 Materials and methods

### 2.1 Human remains

**Figure 1:**
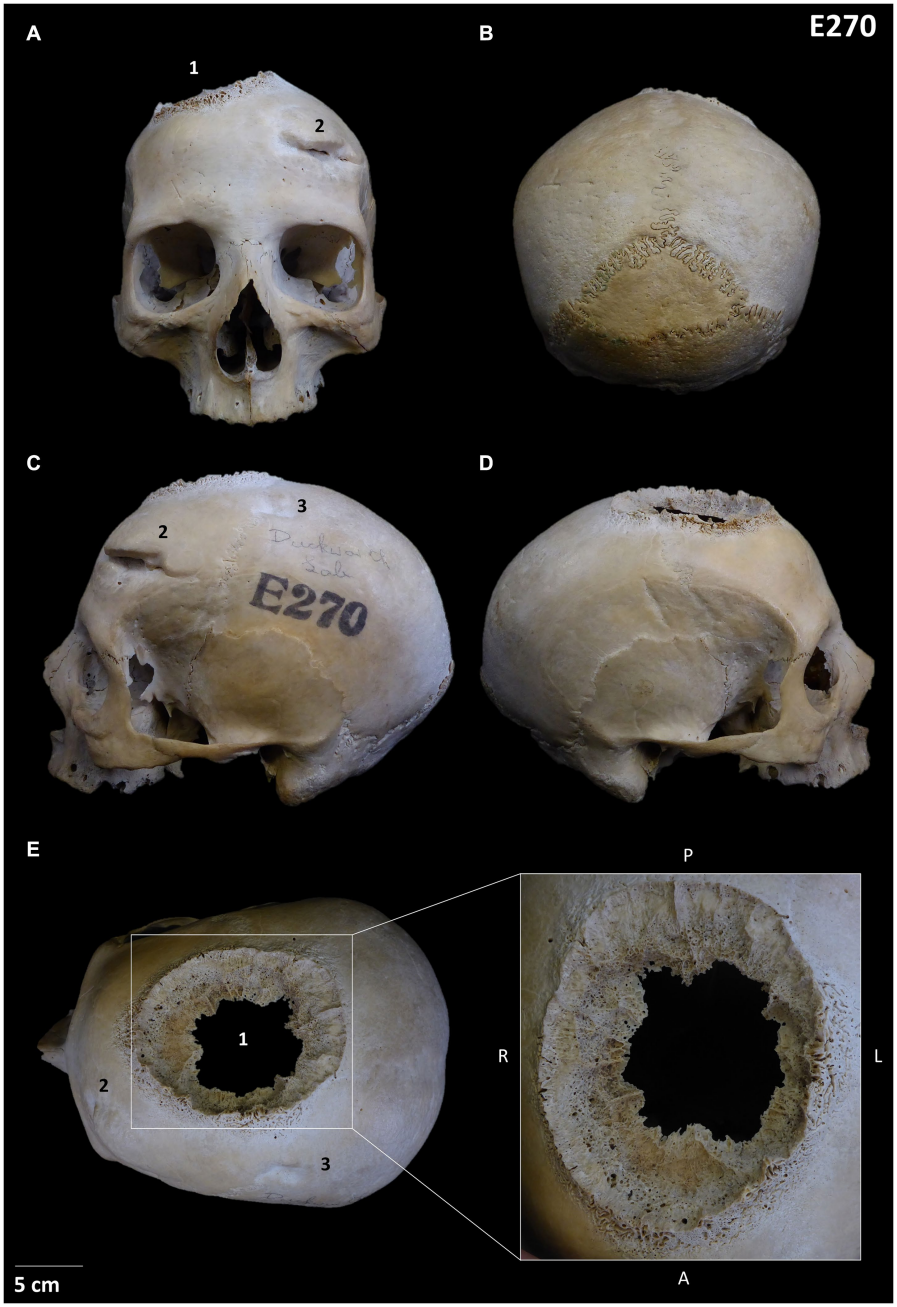
Skull E270: **(A)** Frontal position showing the three lesions; **(B)** Posterior view; **(C)** Left lateral view showing lesions 2 and 3; **(D)** Right lateral view; **(E)** Detail of the neoplastic lesion identified (lesion 1). Other lesions observed are numbered from 1 to 3.

**Figure 2:**
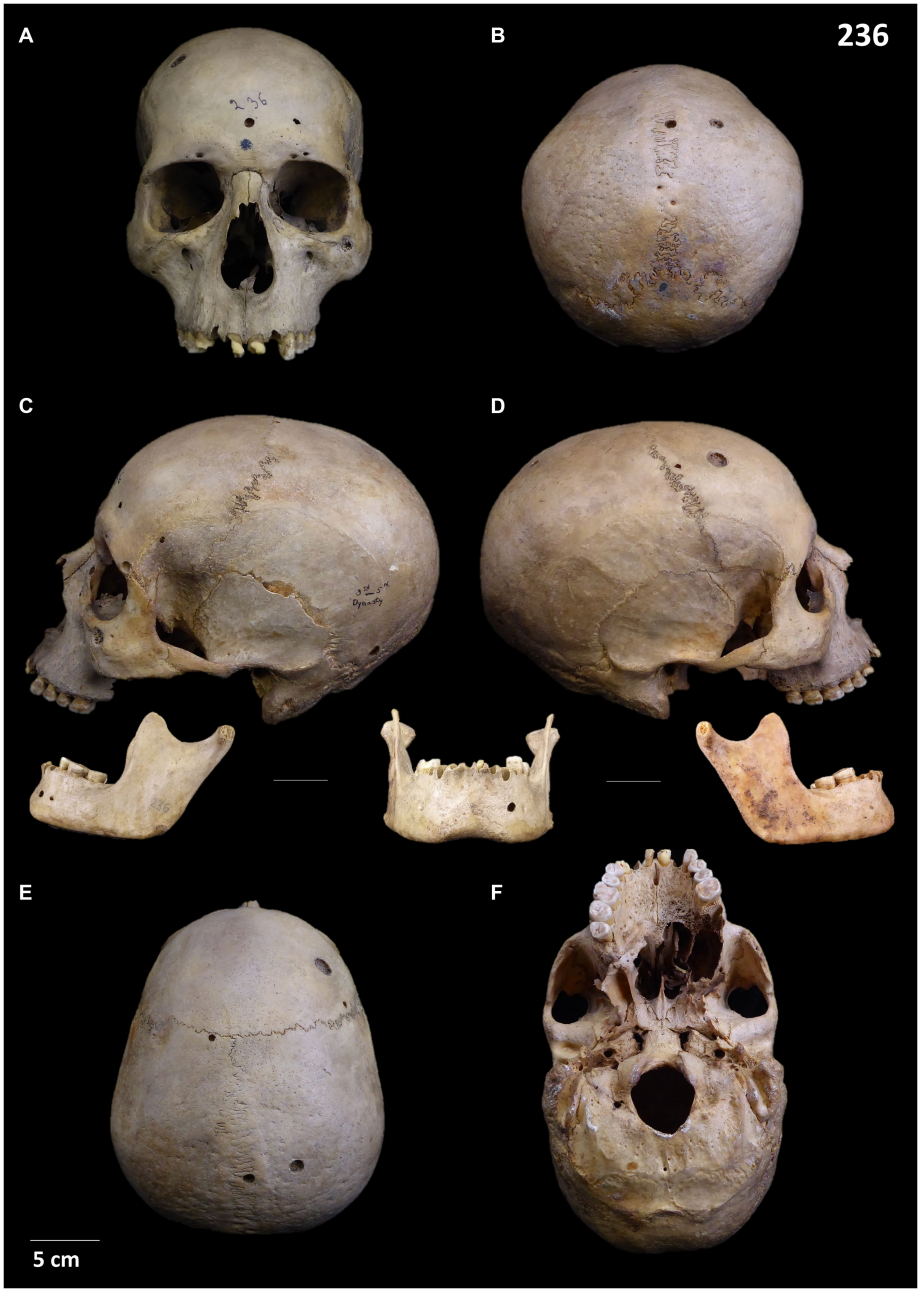
Skull 236: **(A)** Frontal view; **(B)** Posterior view; **(C)** Left lateral view and mandible; **(D)** Right lateral view and mandible; **(E)** Superior view; **(F)** Inferior view.

The two skulls (Accession Numbers E270 and 236) are held at the Duckworth Laboratory (DL) of the University of Cambridge. Both skulls do not include post-cranial bones. As far as we know, there are no records of published studies on skull E270 (Figure 1). The record at the DL shows that skull E270 was found in Giza, Egypt, and dated between 664 and 343 BCE (26–30 Dynasties). On the contrary, skull 236 (Figure 2) was previously analysed by palaeopathologist Wells (21), reporting that the skull dates from 2,686–2,345 BCE, (3–5th Dynasty, Old Kingdom). Wells determined that the skull belonged to an adult male with an age estimated of 30–35 years, and argued that the small perforating skull lesions are the result of malignancy, excluding the options of trauma or infectious conditions (1963: 264). He diagnosed a case of carcinoma of the naso-pharynx with primary destruction of the maxillary, palatal, and pterygoid elements with secondary deposits around the skull.

### 2.2 Anthropological and palaeopathological study

The study of the human remains was conducted at the Department of Archaeology, University of Cambridge. The specimens were characterised anthropologically by carrying out a detailed inventory, measurements, and photographs. The sex estimation was undertaken following Walker in Buikstra and Ubelaker (22) and age estimation using Meindl and Lovejoy (23) for the sutures and Lovejoy (24) for the dental wear analysis. The palaeopathological analysis aimed at a preliminary characterisation of the pathological conditions, but also any potential taphonomic damage. The criteria used to diagnose malignant lesions, including bone destruction (osteolytic process) and bone formation (osteoblastic process), was based on the guidelines provided by Buikstra and Ubelaker (22), Ortner (25), Brothwell (26), and Marques (27, 28). Trauma was examined following White et al. (29), and Roberts and Connell (30). The presence of any potential signs of anthropic modification was analysed following White et al. (29), and the distinction between pathological conditions and postmortem modifications using Haglund and Sorg (31) and Botella et al. (32).

### 2.3 Microscopic approach and micro-CT scanning

The microscopic observation was conducted using a HIROX Digital Microscope HR-2016. The microscope represented an advancement in the characterization of the osteoblastic and osteolytic lesions for a correct diagnosis. In addition, the microscope helped the distinction between taphonomic and pathological damages, and allowed the precise measurement of all the visible lesions on the skulls. In this sense, the evaluation of the microscopic results (along with the pathological analysis) shaped the preliminary description of the bony changes and differential diagnosis of the conditions suffered.

The skulls were analysed with two different micro-Computed Tomography (m-CT) scans. Skull E270 was analysed with a Bruker Skyscan 1,273 m-CT scanner (165 kV, 128 μA, 71.274 μm), whilst 236 was analysed with a Nikon XTEK H 225 ST scanner (130 kV, 100 μA, 81.020 μm). The m-CT scan images were examined with free software, i.e., Dragonfly and DataViewer, through an academic licence. The scans allowed the detection and diagnosis of pathological conditions (e.g., (26, 33, 34)), including malignancy (e.g., (35, 36)) that may not be apparent on the external surface of the bones (36).

## 3 Results

Our anthropological analysis estimated skull E270 to belong to an adult female individual with a skeletal biological age older than 50 years, whilst individual 236 to be an adult male of 30–35 skeletal years (see Supplementary Data). Both of them display pathological lesions described below.

**Figure 3:**
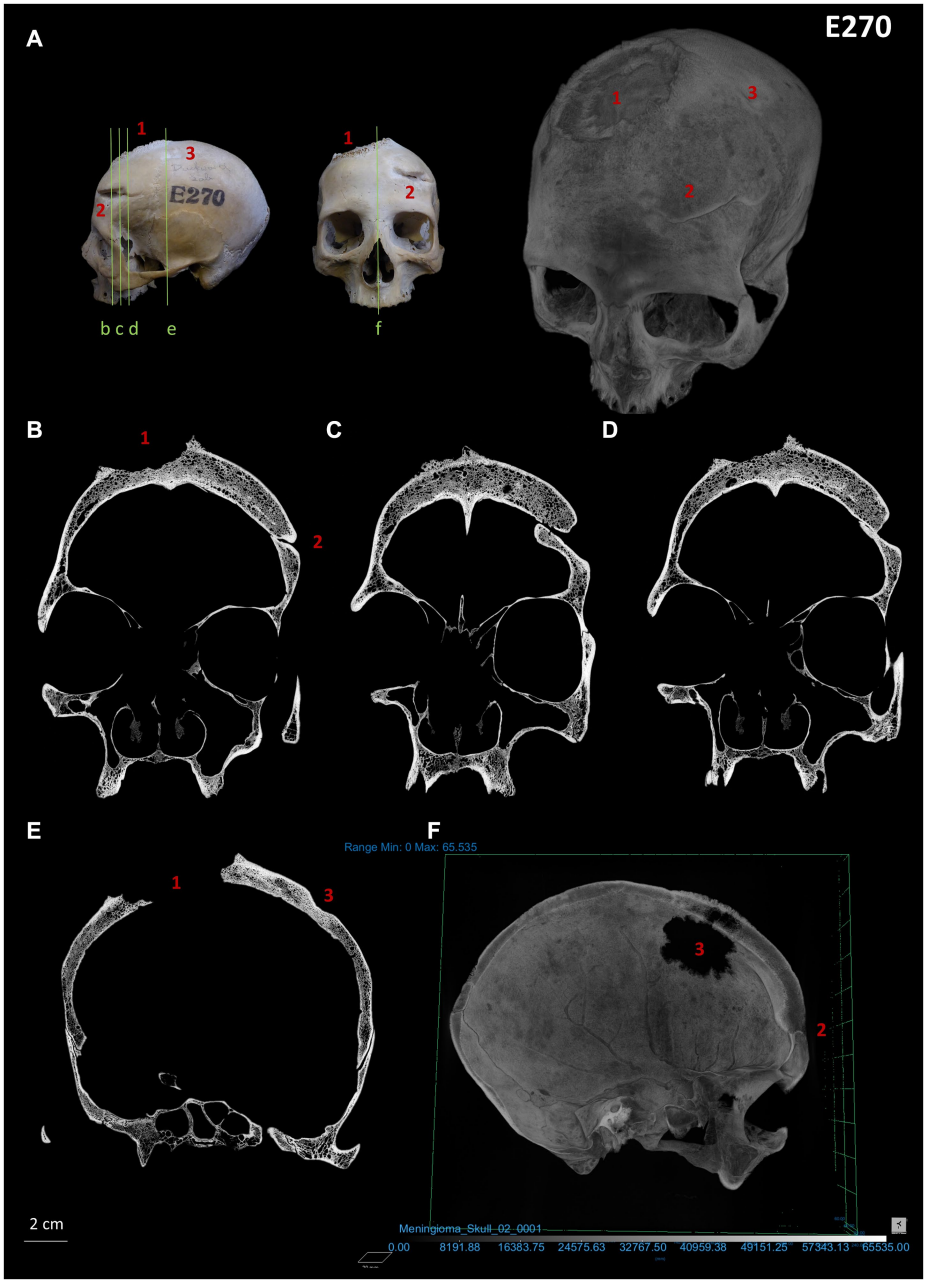
Micro-CT images (coronal plane) and virtual reconstruction of skull E270: **(A)** images showing the different angulations from which the skull was analysed along with a virtual reconstruction showing the three lesions identified. **(B-E)** Lesion 1 displays the presence of a Codman’s triangle, a clear osteolytic destruction (including internal lesions) and aggressive spiculated periosteal reaction (sunburst). Healed traumatic lesions 2 (fracture resulting from sharp trauma) and 3 (blunt force trauma) display periosteal reaction; **(F)** Cross section of the virtual reconstruction of the skull with lesions 1 and 2 visible.

The analysis showed that skull E270 has three main differentiated lesions. The first one is an irregular big-size orifice located between the right frontal and parietal bones and can be defined as a mixed osteological reaction involving an osteolytic and sclerotic process with a well-defined transition zone (lesion 1 in Figure 1 and Supplementary Data Figure 1). Macroscopically the lesion displays four differentiate areas: (i) the inner zone with a jagged-like contour of the inner and outer table with a moth-eaten morphology; (ii) an hyperostosis surrounding the diploe and a well-defined transitioning zone from the inner to the outer area; (iii) an irregular radiant sunburst [as described by Marques (27, 28)] forming a massive spiculated periosteal reaction in alignment with the cortex; and (iv) a peri-reactive osteoblastic reaction surrounding the lesion with a clear development on the frontal region. A microscopic approach has helped in the characterisation of these areas (Supplementary Data Figure 1). The internal m-CT scan showed the presence of a Codman’s triangle, in addition to internal osteolytic lesions (Figure 3; Supplementary Data Figure 2). The pathology is consistent with a neoplastic lesion, and specifically a malignant neoplasm involving bone marrow lesions, cortical bone destruction and aggressive periosteal reaction. Although this type of lesion is consistent with a primary osteosarcoma or a meningioma, a differential diagnosis is provided as Supplementary Data.

Another observed lesion on skull E270 is a healed sharp-force antemortem weapon-related trauma located on the left side of the frontal bone (lesion 2 in Figure 1), indicative of a potential frontal interpersonal violent event using a sharp-edged blade instrument. This lesion is consistent with a head injury resulting in a deep severe wound, potentially involving compression and torsion, that dislodged and sharply chipped out an area of the anterior frontal bone and produced a fracture with a clean edge towards the posterior area. Internal skull observation reveals bone displacement of the vault due to the depressed skull fracture resulting in inwards crushing (Figures 1A, 3C–D). In addition, a second regular round traumatic injury (lesion 3 in Figure 1C), is located on the left side of the parietal bone, and can be described as a depressed skull fracture with sinking of the cranial vault due to blunt force trauma. Interestingly, despite these injuries might have been produced simultaneously, the individual survived given the well remodelling of the wounds edges indicative of healing (see also bone remodelling from internal m-CT structure analysis in Figure 3). A microscopic approach has revealed an additional healed fracture line with no displacement that is located anatomically in association with neoplastic lesion 1 (Supplementary Data Figures 1D, 3).

Concerning skull and mandible 236, the specimens display osteolytic and osteoblastic lesions consistent with primary and secondary neoplasms. The main lesion can be characterised as a big-sized irregular osteolytic lesion with a moth-eaten morphology on the palate, showing new bone formation around it (Figure 2F; Supplementary Data Figure 4). Macroscopic, microscopic and internal observation confirms the sclerotic process. In addition to this lesion, the skull and the mandible display multiple scattered foci destruction of the bone surface. These pathological lesions are small, rounded in shape, circumscribed with well-defined edges, clear margins, and bone reaction around them. We have registered around 30 lesions displaying such features, in addition to 4–5 small round internal lytic lesions identified through m-CT (Supplementary Data Figure 5). These lesions can be distinguished given its characteristics from the small bone damage with sharp edges, uneven borders and no bone reaction which are likely to be taphonomic damage. Both types of lesions (pathological vs. postdepositional) display clear different features (Supplementary Data Figures 6, 7). The pathological lesions are consistent with a potential metastatic carcinoma (secondary neoplasms), with a primary neoplastic lesion restricted to the palate as a nasopharyngeal carcinoma. For a detailed differential diagnosis see Supplementary Data.

**Figure 4:**
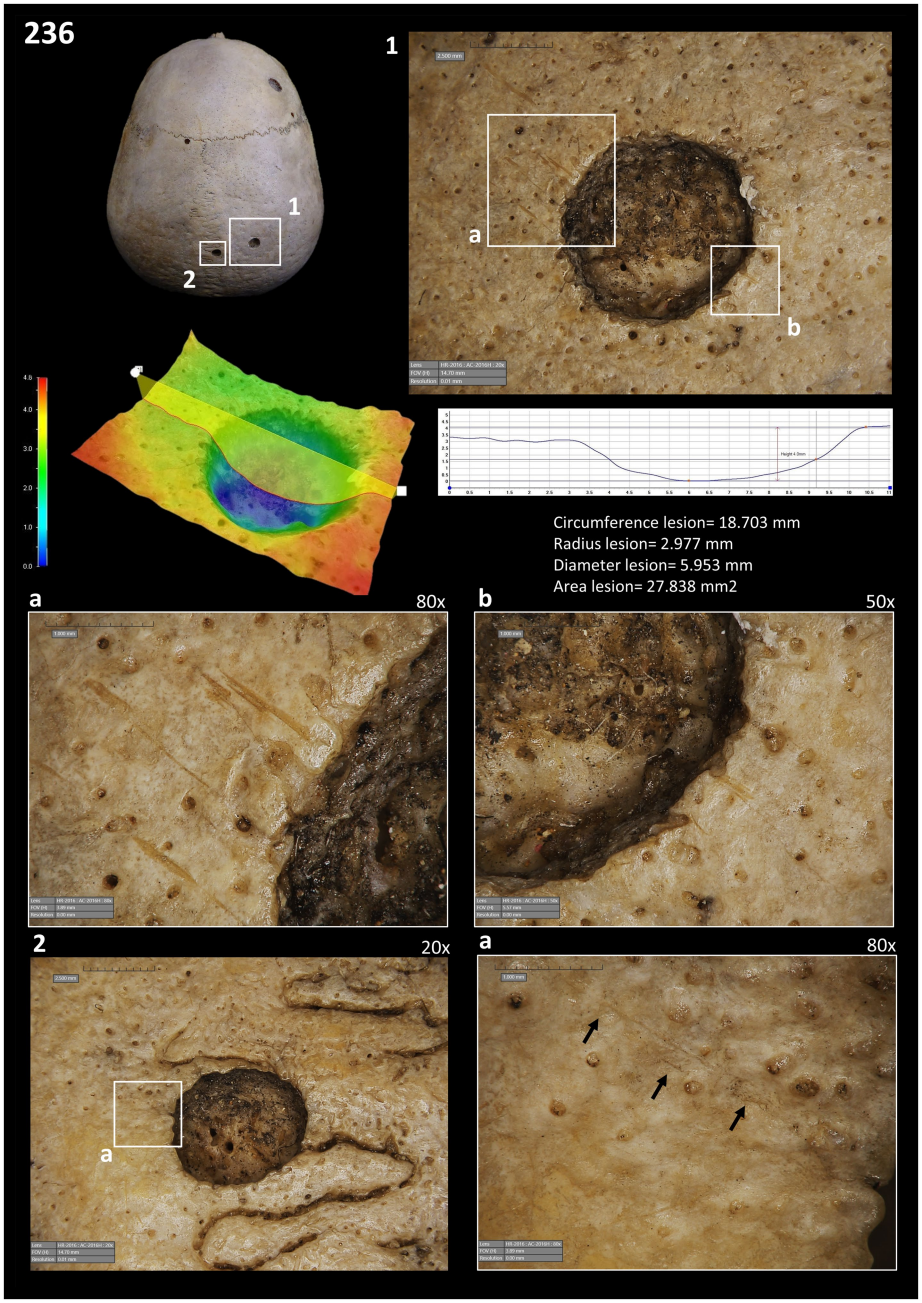
Images of the cut-marks close to the two lesions taken with the HIROX Microscope: (1) images showing the location of the lesions with cutmarks; depth of lesion 1; cut marks of lesion 1 (a and b). (2) images showing the second lesion with evidence of the cut-marks.

Additionally, our microscopic approach revealed the presence of clear linear small perimortem cutmarks in association with the small rounded lytic lesions (secondary tumours) (Figure 4). These anthropogenic marks are located in clear superposition of two lesions on the posterior zone of skull 236, and are described as V-shaped parallel linear marks with internal microstriation conforming groups defining the same direction in different parts of the lesions. The marks display features consistent with anthropogenic modifications on fresh bone (perimortem stage) such as well-defined Hertzian cones, and no bone remodelling or healing has been observed (antemortem stage).

## 4 Discussion and conclusions

The study of medical care and surgical intervention is a complex and challenging subject in palaeopathology, especially for prehistoric and early historic periods (37). Despite this challenging issue, isolated and inconclusive evidence amongst the bioarchaeological record states that potential surgical practises might have been practised during the Middle and Upper Palaeolithic [e.g., (38, 39)] and Neolithic [e.g., (40, 41)], and wound-care and other-regarding behaviour even earlier [e.g., (42, 43)]. In this context, it becomes clear that caring for others, including wound-care, is a key behaviour amongst humans that has also been observed in non-human primates (e.g., 44). However, evidence of caring behaviour and surgical practises is still a challenging scientific frontier within palaeopathology, and the cases discussed here exemplify this boundary in our understanding of medical and health care practises in ancient Egypt.

Ancient Egyptian Civilization has provided outstanding written and bioarchaeological evidence of medical advancement in antiquity regarding infections, traumatisms, and other conditions. Skull E270, provides evidence in such direction, as the healed cranial injuries described are indicative of survival for the individual and some kind of treatment and degree of post-traumatic care. Beyond that, as stated for other weapon-related bioarchaeological cases, the degree of brain function impairment is not possible to infer solely from the skeletal remains (45). However, palaeopathology has assumed that severe skeletal trauma might be linked to a group/social response in terms of care and wound-related treatment (16, 46). In this sense, and given the severe nature of the head trauma (one of them clearly with a weapon-related origin) described here for E270, we assume some kind of injury treatment, in light of ancient Egyptian medical knowledge mentioned.

Interestingly, ancient Egyptian interventions, despite cultivating such medical knowledge regarding several conditions and even potential neurosurgical therapies (13), including traumatism management as seen, malignancy was a clear boundary regarding both diagnosis and treatment. Skull E270 and 236 display pathological lesions consistent with malignancy. Actually, 236 represents one of the oldest known cases of ancient Egyptian cancer, jointly with others from the Old Kingdom [see Supplementary Data in Hunt et al. (15)]. Whilst there is no evidence regarding the cause of death for both individuals, the advanced stage of their malignant conditions suggests a potential link to mortality. This illustrates a clear differential medical limit during ancient Egypt when approaching the treatment and caring management of both malignancies and skeletal trauma. Nevertheless, although neoplasms were a clear medical frontier, skull 236 reveals new insights on a potential exploratory phase amongst medical practise concerning neoplastic lesions. As seen, reliable perimortem cutmarks on the bone surface have been identified in clear association with the metastatic lesions on the posterior cranial region. The position of the marks, running through two of the lesions with a clear associated start and end at both sides of the lytic lesions (stopped by the margins of the pathologies), suggest some kind of perimortem anthropic intervention given that they were generated on a bone in fresh condition. Although this might indicate medical surgical exploration or an attempt of care or treatment, our study has a clear limitation in the identification of the timing of the cutting. Although they are perimortem, they might also indicate a postmortem manipulation of the corpse. In turn, this might also indicate a postmortem exploration of the tumoural pathology. What is clear, is that the mummification process, when affecting cranial skeletal structures, does not involve cutmarks on the posterior region of the parietal (47, 48). Also, these marks do not resemble postdepositional taphonomic marks such as trampling or similar [see (49)].

As side observations, it is interesting to highlight that the primary tumour and a healed fracture line observed on skull E270 has an anatomical geographic association (Supplementary Data Figure 3). As observed microscopically, a linear healed fracture runs through the neoplastic lesion parallel to the coronal suture. Although clinical association between fractures and tumorigenic lesions has been suggested, specially for osteosarcoma [e.g., (50, 51)], clinical data do not relate trauma as a causing factor (51).

It is also worth mention that the present study includes some main limitations concerning the regressive diagnosis given the nature of the sample, and analysis scope. First, the sample relies on incomplete skeletal remains, which restrict the picture of the conditions suffered by the two individuals. Second, the analysis only includes two individuals, thus limiting the possible inferences regarding ancient cancer in Egypt. And third, molecular analyses were not implemented, which could have enhanced the completeness of the analysis. Future paleopathological studies, with the aim of understanding of the historical prevalence and causation of cancer, will need to integrate such molecular scope, when possible. Such approach will not only broaden our understanding of ancient cancer mechanisms but also improve the accuracy of past disease diagnosis and its socio-economic implications. However, despite these challenges and limitations, our research highlights in turn the potential of non-invasive methods in paleopathology and paleo-oncology research.

Other interesting observations amongst our case studies is the female sexual estimation of skull E270 in relation to the antemortem weapon-related traumatic lesions observed. As an example, the healed sharp force trauma was produced by a sharp instrument [e.g., see potential Egyptian weapon repertoire in (13): 197], and given its location might have been produced in the context of a face-to-face frontal attack, with all characteristics of an interpersonal violent event involving a right-handed perpetrator [e.g., (52)]. Therefore, given the characteristics described we discard an accidental related-injury in favour of an interpersonal violent event, at least for lesion 2. In this sense, it is interesting to observe such type of wound in a female individual, as demographically, violence-related skull injuries are associated with males in most chronologies [e.g., (53)], including ancient Nubian and Egyptian cases [see (54) and references therein]. However, this provides challenging bioarchaeological observations concerning gender-related activities, such as involvement in warfare. Actually, some authors provide skeletal-related data that suggests a similar ancient Egyptian male–female ratio of traumatic injuries in the context of political and social conflict (55). Nevertheless, the skeletal pattern of traumatic violent-related injuries, and its behavioural and social meaning, is an open debate for many historical periods [see (56, 57)].

In conclusion, our cases contribute to an increasing perspective of a higher prevalence of cancer in past human populations [see (15, 58); Marquez et al., 2022], by providing and discussing two cases, one of which represents one of the oldest known cancers from ancient Egypt. Also, our study shows the importance of re-analysing using new techniques and different scope palaeopathological cases from museums and university collections with the aim of providing new insights into past societies, including health issues. In this sense, our study has implemented micro-internal bone characterisations using micro-CT scans, a necessary analytical approach to relate the observation of internal lytic lesions and a diagnose of malignancy amongst archaeological skeletal remains as suggested by Mitchell et al. (59). In this sense, paleoradiology is key to undertake non-invasive regressive diagnosis amongst cases involving ancient remains. In addition to detailed macroscopical observations, we acknowledge the implementation of a microscopic characterisation of the bone and lesion surfaces. Such observations have led us to a better diagnosis of neoplastic pathology, specifically the lesion margins. Furthermore, therapeutic practises or medical exploration have been interpreted as a result of such micro-approach, contributing to the understanding of caring behaviour in the context of the early history of medicine.

## Data availability statement

The raw data supporting the conclusions of this article will be made available by the authors, without undue reservation.

## Ethics statement

The studies involving human remains were reviewed and approved by the Duckworth Laboratory and the Department of Archaeology (University of Cambridge). Written informed consent for participation was not required for this study in accordance with the national legislation and the institutional requirements.

## Author contributions

TT: Conceptualization, Data curation, Investigation, Methodology, Writing – original draft, Writing – review & editing. AI: Conceptualization, Investigation, Writing – original draft, Writing – review & editing. EC: Conceptualization, Data curation, Formal analysis, Funding acquisition, Investigation, Methodology, Project administration, Resources, Supervision, Writing – original draft, Writing – review & editing.
