# Supplementary material for: Case report: Boundaries of oncological and traumatological medical care in ancient Egypt: new palaeopathological insights from two human skulls
Source: Front Med (Lausanne). 2024 May 29;11:1371645. doi: 10.3389/fmed.2024.1371645 (PMC11167094; doi:10.3389/fmed.2024.1371645)
Supplement: Supplementary file 1 [file Data_Sheet_1.pdf]

Supplementary Data

Anthropological characterisation

Sex estimation

Although our analysis relies solely on the skulls, we can still provide a very accurate but not totally exhaustive sex estimation.

Our analysis adopted the standards by Walker in Buikstra and Ubelaker (1994) to estimate sex using the skull, which is currently the most used among bioarchaeologists (Quinn, 2017). The Walker standards characterise a five-point scale, namely the shape and size of the nuchal crest, mastoid process, supraorbital margin, supraorbital ridge/glabella, and mental eminence. Sex estimation is anthropologically carried out using a rating system of the different morphological characteristics, rather than a distinct Female or Male. This means that individuals are assigned one of the five sex classifications: Likely Female; Probable Female; Indeterminate, Probable Male, or Likely Male. The more gracile features are closer to the *Likely Female*, while the more robust and larger features are toward the *Likely Male*.

The biological sex of the skull E270 results in “Probable Female.” The examined five-point scale shows characteristics that are typical of a female individual. The nuchal crest shows that the external surface of the occipital is relatively smooth with a slight bone projection. The mastoid processes are relatively small, projecting only a small distance. The supra-orbital margin, i.e., the margin of the orbit, presents an extremely sharp border. Finally, the glabella shows the contour of the frontal as smooth with little projection at the midline. The last criterion, i.e., the mandible mental eminence, is unclassified (the mandible is not present). In short, the overall skull assessment suggested that the biological sex of the individual to whom skull E270 belonged is probably female.

The analysis of the biological sex of the skull 236 results in “Probable Male.” The five characteristics examined to determine the sex are typical of a male individual. The nuchal crest shows that the external surface of the occipital projects a considerable distance from the bone, forming a well-defined bony ledge. The mastoid processes of both sides are massive in size and their lengths and widths are several times bigger than that of the external auditory meatus. The glabella is relatively prominent with a rounded and loaf-shaped projection. The supra-orbital margin presents a thick and rounded margin with a curvature. Finally, the mental eminence shows a massive projection that occupies most of the anterior portion of the mandible. These characteristics suggest that the individual to whom the skull belonged is a male.

Age determination

The sutures of the cranium from Skull E270 are fully closed, shown by their smooth and continuous surface, suggesting that the individual reaches adulthood (While and Folkens, 2005). Furthermore, even though the teeth are not preserved, the sockets of three left molars (therefore including the third

molar, also known as wisdom teeth, normally associated with adulthood) manifest resorption of the alveolus, meaning that the teeth were lost during life, and the bone had time to heal itself (O'Connell, 2004; White *et al.*, 2011; Nikita, 2016). This means that the individual E270 is probably an old adult (White *et al.*, 2011). In short, the analysis led to the conclusion that the individual clearly reaches adulthood, and that, based on the teeth, is probably above the age of 50.

The sutures of the cranium from Skull 236 show a smooth and continuous surface, leading to the conclusion that the sutures are fully closed, and that the individual reaches adulthood (While and Folkens, 2005). The teeth of the maxilla and mandible are compared with those of the standard reference. The teeth of the maxilla are estimated to age around 25-30 years, whilst the teeth of the mandible are around 35-40 years. Therefore, the analysis led to the conclusion that the individual 236 clearly reaches adulthood, and that, based on the teeth, he is probably around the age of 30 (between young and middle adulthood).

Differential Diagnosis

- **Skull E270**

Osteosarcoma

Osteosarcomas (OSs) are rare types of malignant neoplasms when related to the cranial vault and frequently metastasize to the lungs and liver (Ortner *et al.*, 2012; Marques, 2019). OS are characterised as a single lesion, which can become a large perforation, surrounded by massive periosteal reactions (Marques, 2019). The periosteal reactions can form either coarse irregular "sunburst" speculation, multiple layers ("onion skin"), and/or Codman's angle (Cole *et al.*, 2022; Strouhal *et al.*, 1997; Gorlick and Khanna, 2010). Specifically, the sunburst speculation is a fundamental feature in recognizing this neoplasm (Brothwell, 2012; Pechenkina *et al.*, 2019). Therefore, the single large lesion, typical "sunburst" speculation, and the Codman's triangle seen in skull E270 suggest the diagnosis of OS.

Meningioma and MeningioSarcoma

Meningiomas (MGs), which are predominantly benign tumours that arise in the meninges, may lead to a bony response that forms radiating spicules in the cranial vault similar to Oss (Pechenkina *et al.*, 2019; Marques, 2019; Cook and Danforth, 2022; Cucu *et al.*, 2019; Shang and Trinkaus, 2008). However, the main differential diagnostic features of meningiomas are that their lytic lesions are normally small in size and show slow growth of new bone (Marques, 2019). In addition, speculated reactions are characteristics, especially of OS rather than MG (Cucu *et al.*, 2019; Strouhal *et al.*, 1997). We can put aside the diagnosis of malignant meningioma takening in account several cases of the paleopathological record. Therefore, even though MG can be a potential diagnosis for the lesion seen in skull E270, thus hard to completely reject it, the aggressive nature of the lesion, its large size, and the sunburst reactions does not exclude OS in favour of MG.

Osseous Hemangioma

Haemangiomas (HMGs) are benign tumours of endothelial cells and can produce osteolytic lesions (along with the "honeycomb" or "sunburst" pattern) that mimic the appearance of OS (Marques, 2019; Wu *et al.*, 2011). However, hemangiomas normally display lesions that are thinner compared to those seen in malignant tumours (Marques, 2019). Therefore, even though HMG can be a possible diagnosis,

the sum of all the characteristics of the lesion observed in skull E270 makes OS and MG, again, more likely.

Metastatic Carcinoma

Metastatic carcinomas, secondary malignant tumours, can produce osteolytic and osteoblastic reactions similar to those seen in osteosarcomas (Marks and Hamilton, 2007; Baraybar and Shimada, 1993; Marques, 2019; Binder *et al.*, 2014). However, the main differential diagnostic features are that single metastases are extremely rare and normally do not develop the "sunburst" pattern (Binder *et al.*, 2014). Therefore, the fact that skull E270 presents a single lesion and the sunburst pattern argues against metastatic carcinoma as a possible diagnosis for the lesion in question.

- **Skull 236**

Metastatic Carcinoma

Metastatic carcinomas can produce osteolytic (75%), osteoblastic (15%), or a combination of both skeletal responses (10%) (Baraybar and Shimada, 1993). The osteolytic lesions can vary both in size (ranging from 3 to 47) and shape (Marques, 2019; Lieverse *et al.*, 2014). Based on the aggressiveness of the disease, the lesions can be either well-circumscribed (geographic) or poorly defined (moth-eaten) (Marques, 2019). In cases of metastatic carcinomas, m-CT scans usually reveal several more internal lesions (Binder *et al.*, 2014). Skull 236 shows no aspects of lesion morphology or anatomical distribution that argue against a diagnosis of metastatic carcinoma. The origin of the metastases is probably the palate, leading to the hypothesis that this is a nasopharyngeal carcinoma.

Multiple Myeloma

Multiple myelomas (MM), which are primary cancers caused by the gathering of plasma cells within the hematopoietic bone marrow, produce several destructive lesions that can mimic those caused by metastatic carcinoma (Lieverse *et al.*, 2014; Marks and Hamilton, 2007; Ortner, 2003). The main diagnostic features are the size, shape, and distribution of the lesions (Binder *et al.*, 2014). MMs produce lesions that are quite small, uniform in size (ranging from 3 to 10 mm), rounded with effaced edges, smooth borders, regular and dense distribution of the lesions (Strouhal, 1991; Ortner, 2003; Marks and Hamilton, 2007). In addition, MMs typically produce osteolytic lesions (Schultz *et al.*, 2007; Marques, 2019; Lieverse *et al.*, 2014). The lesions on skull 236 are variable in shape, have well-defined margins, are distributed irregularly around the skull, and present new bone formation around the palate, thus arguing against MM as a diagnostic possibility for the lesions in question.

Leper, Trepanomatosis and Mycosis Infections

Several fungal infections (mycotic disease) can produce osteolytic lesions that can resemble the appearance of metastatic carcinoma (Rubini *et al.*, 2022). However, the main diagnostic features are that fungal infections cause lesions with the appearance of resorption of the bone rather than space-occupying lesions seen in metastatic carcinomas (Binder *et al.*, 2014). In addition, fungal infections rarely create new bone, and if they do, it is normally circumferential periosteal formation surrounding the lesions (Lieverse *et al.*, 2014). The lesions observed on skull 236 do not conform to the features associated with mycotic fungal infection, thus making mycotic fungal infection an unlikely hypothesis.

Taphonomic damage and trepanation

Taphonomic processes can produce small rounded holes on bones that mimic the damages created by metastatic lesions (Lieverse *et al.*, 2014; Strouhal, 1991). Taphonomic damages can be distinguished from pathological conditions by analysing several characteristics, namely the presence of inchoate

lesions within cancellous bone; the presence of furrows and foraging galleries; and the absence of new bone formation around and/or near the lesions (Lieverse *et al*., 2014; Huchet *et al*., 2011). The analysis thus confirms that even though some lesions were clearly caused by taphonomic damages, others show clear evidence of pathological conditions.

Given the pathological characteristics and the absence of any anthropic feature related to the perforation lesions, we discard trepanation, cutting or drilling of openings on the skull which would be revealed by mechanical opening of the cranium among other diagnostic features (see Verano, 2006).

Detailed paleopathological characterization - Images

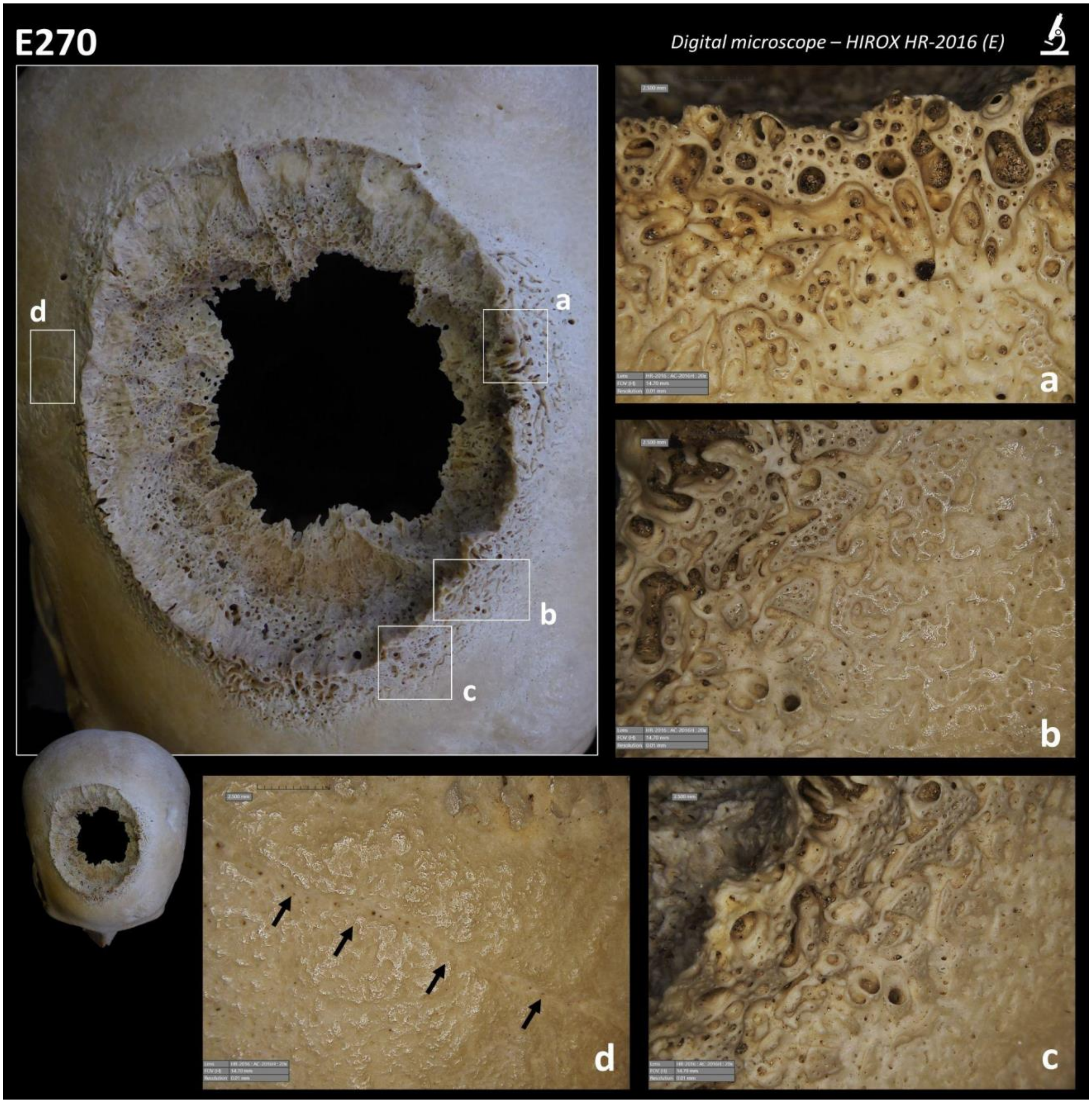

Supplementary Data Figure 1. Images taken with HIROX Microscope: **a-c**) Images showing osteoblastic reactions; and **d**) Linear healed fracture close the neoplasm lesion**.**

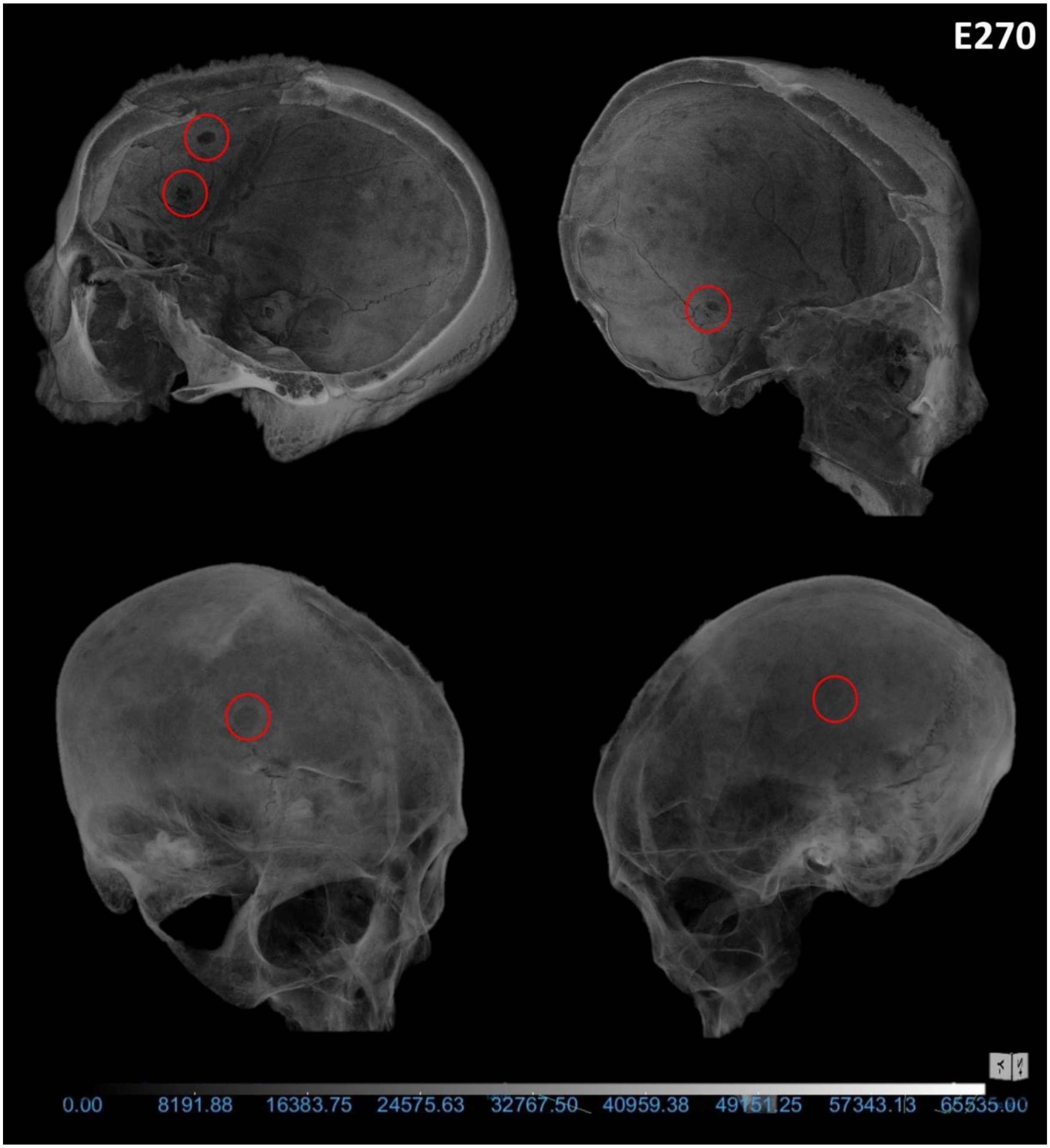

Supplementary Data Figure 2. Virtual reconstruction of micro-CT images of skull E270 with internal lytic lesions visible and highlighted.

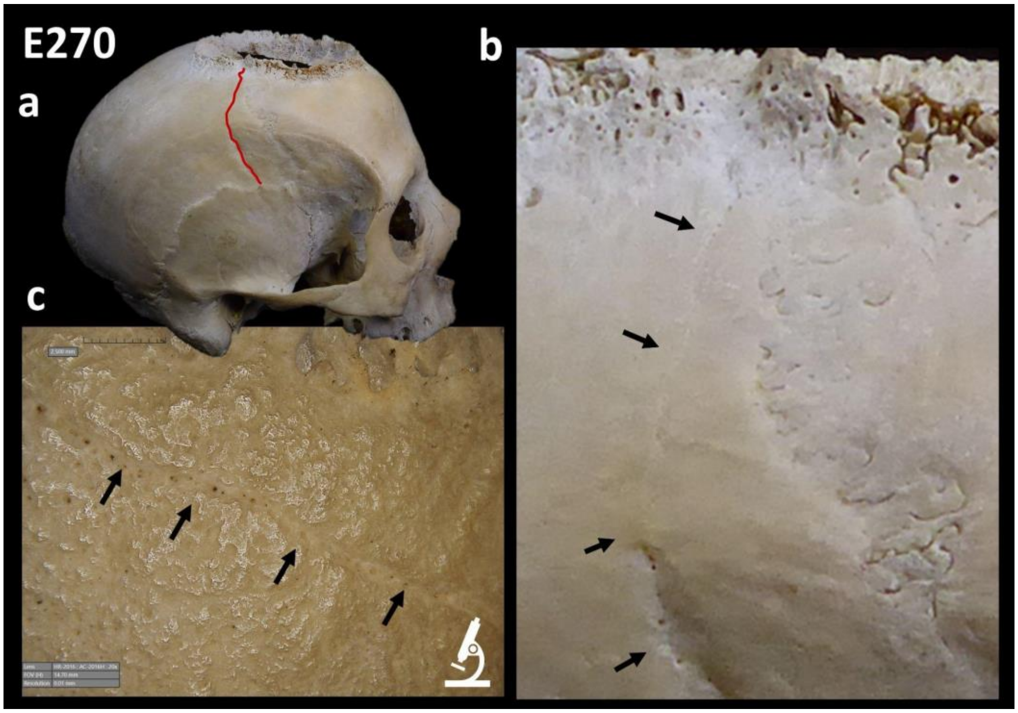

Supplementary Data Figure 3. Linear healed fracture close the neoplasm lesion.

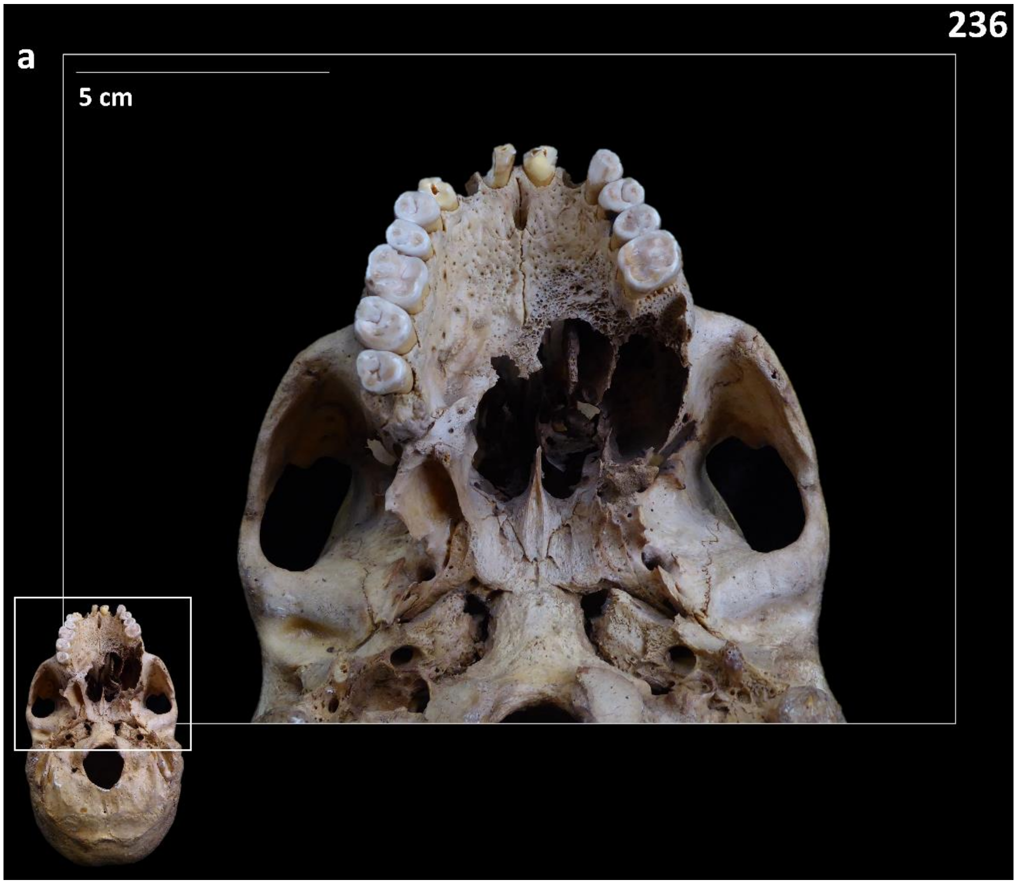

Supplementary Data Figure 4. Lytic lesion on the palate.

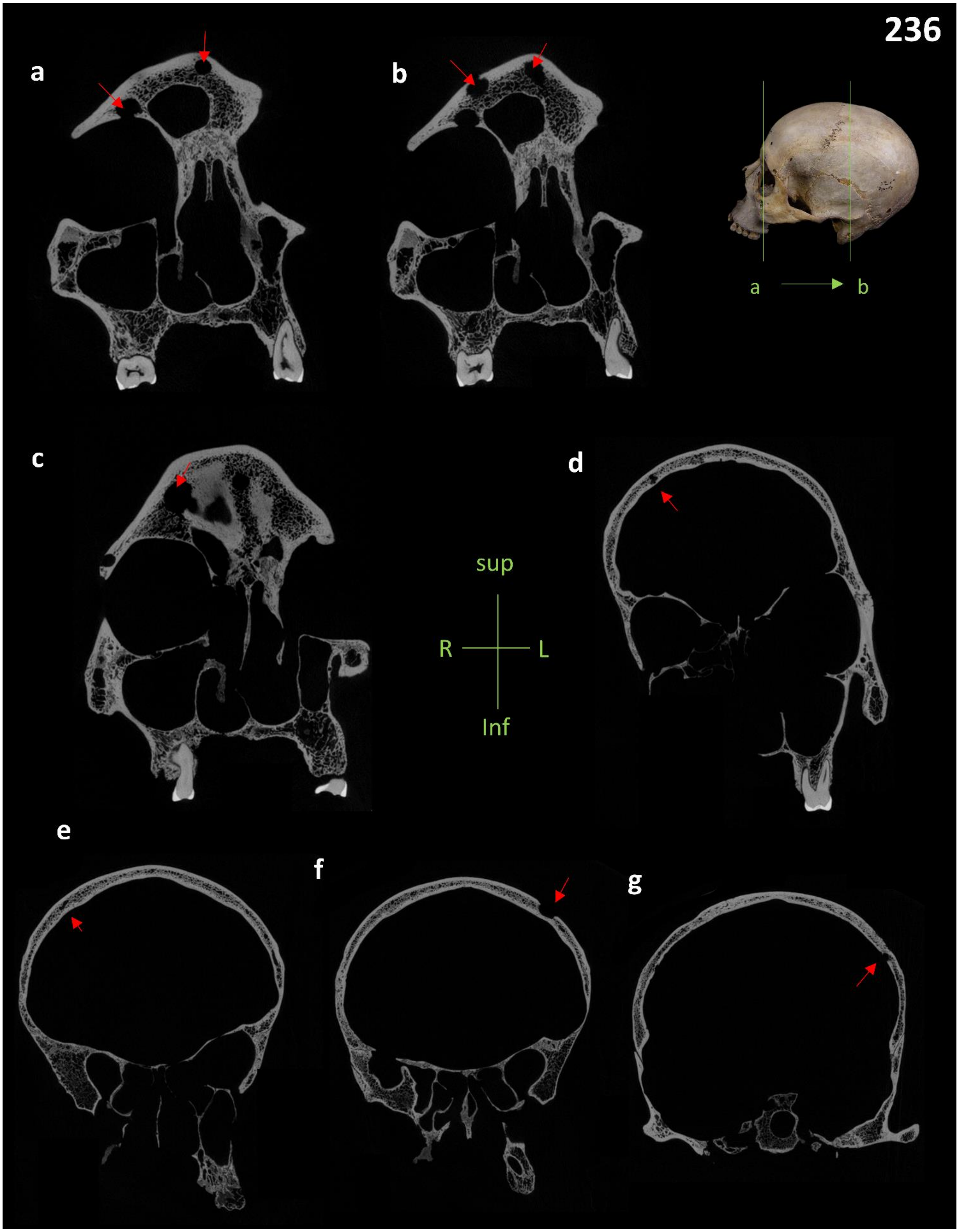

Supplementary Data Figure 5. Micro-CT images of skull 236 examined using DataViewer and Dragonfly. Reconstruction of virtual images (coronal plane) that shows the internal lesions indicated by the red arrows.

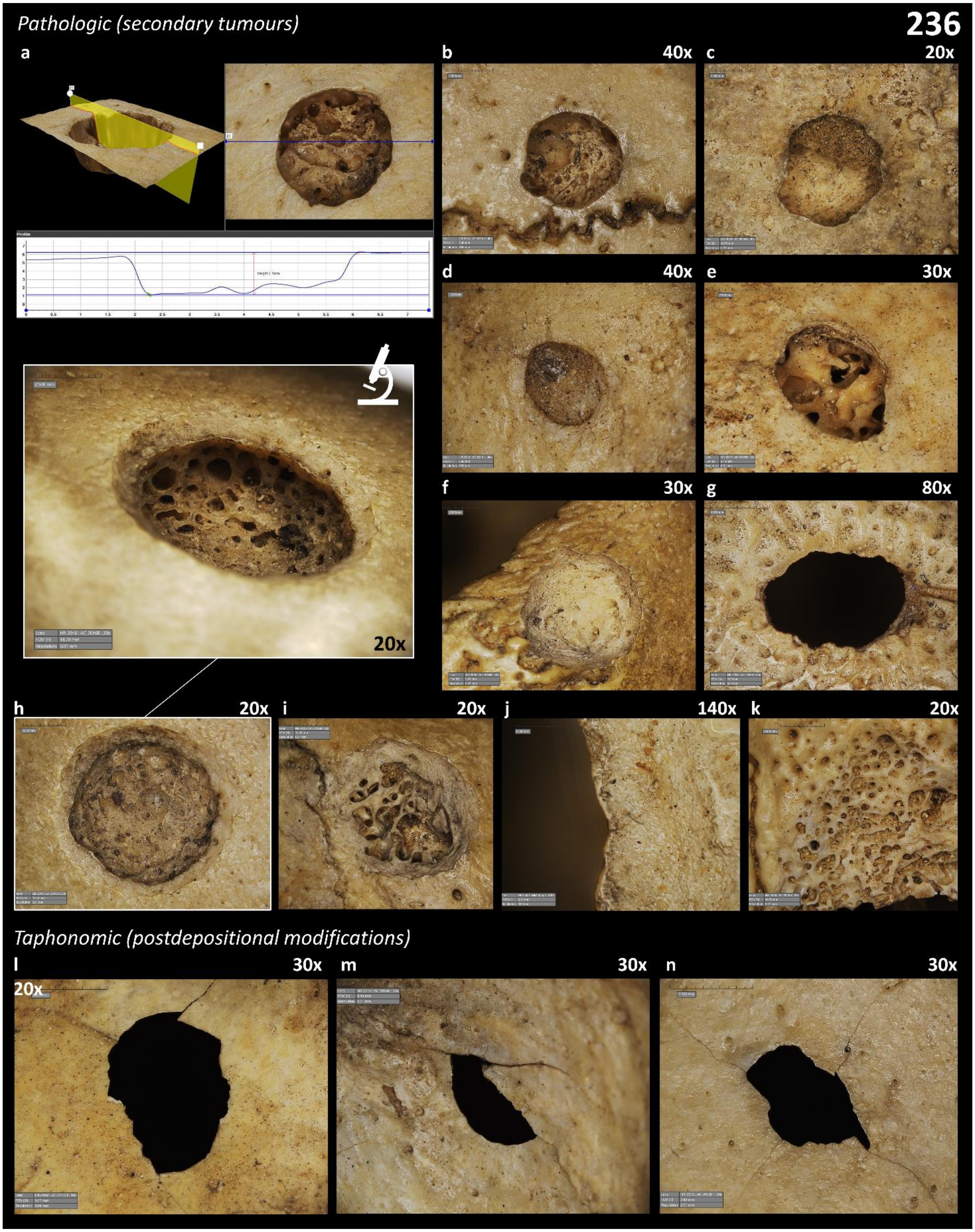

Supplementary Data Figure 6. Images of the lesions taken with the HIROX Microscope showing: **a**) Example of measurement of the depth of one of the pathological lesions; **b**- **i**) Pathological lesions; **j**) Sclerotic process; **k**) Sclerotic process on the margins of the primary tumour site on the palate; **l-n**) Taphonomic postdepositional lesions.

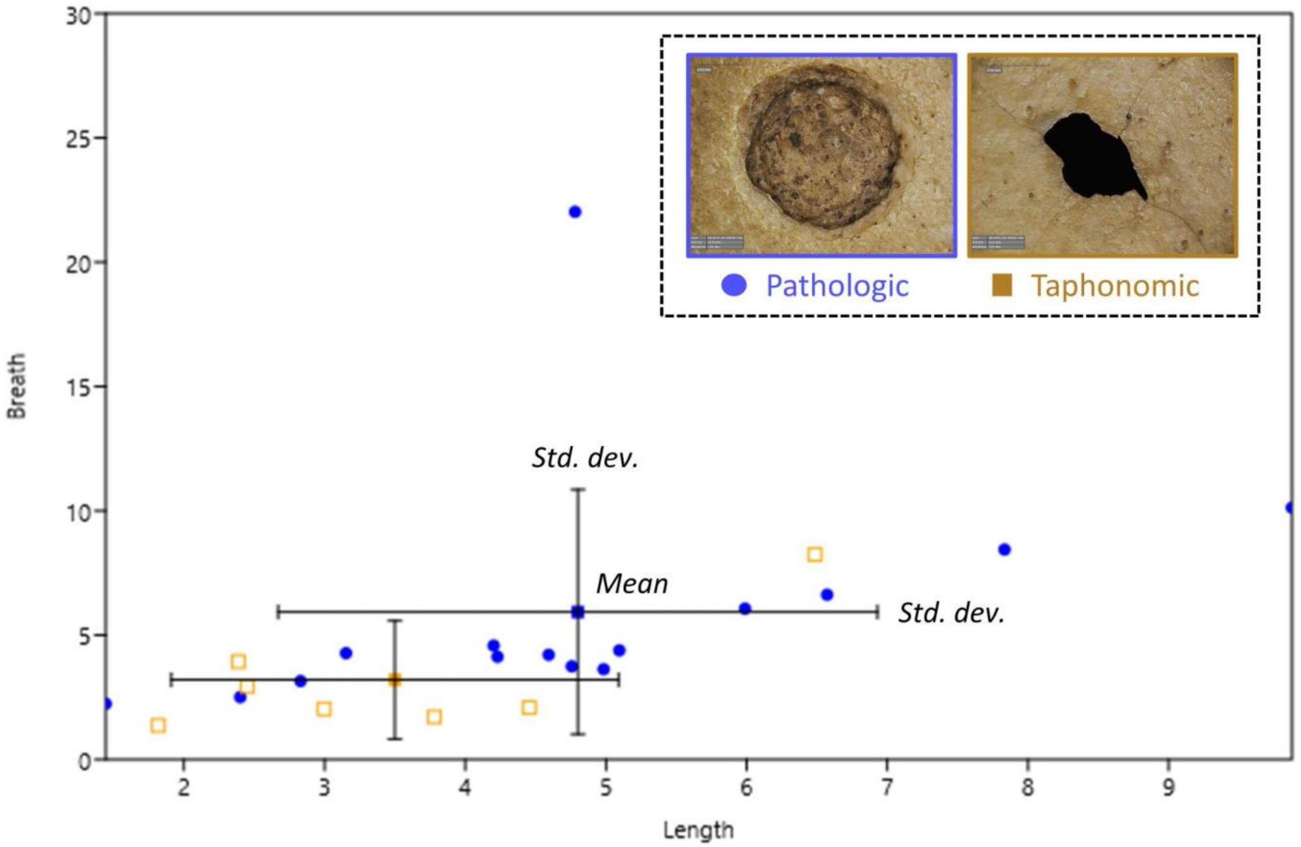

Supplementary Data Figure 7. Differences in measurements (breath and length) between pathologic round lytic lesions and taphonomic postdepositional damage observed on skull 236.

References

Baraybar, J. P., and Shimada, I. (1993). A possible case of metastatic carcinoma in a Middle Sican burial from Batan Grande, Peru. *International Journal of Osteoarchaeology, 3*(2), 129-135.

Binder, M., Roberts, C., Spencer, N., Antoine, D., and Cartwright, C. (2014). On the antiquity of cancer: Evidence for metastatic carcinoma in a young man from Ancient Nubia (c. 1200BC). *PloS one*, *9*(3), e90924.

Brothwell, M. (2012). Tumors: Problems of Differential Diagnosis in Paleopathology. In Grauer, A.L., (eds), *A Companion to Paleopathology*, pp. 420-433. Hoboken: John Wiley & Sons.

Buikstra, J.E. and D.H. Ubelaker. (1994). *Standards for Data Collection from Human Skeletal Remains*. Fayetteville, Arkansas Archaeological Survey Research Series No. 44.

Cole, G., Taylor, G. M., Stewart, G. R., and Dawson-Hobbis, H. (2022). Ancient DNA confirmation of lepromatous leprosy in a skeleton with concurrent osteosarcoma, excavated from the leprosarium of St. Mary Magdalen in Winchester, Hants., UK. *European Journal of Clinical Microbiology & Infectious Diseases*, *41*(11), 1295-1304.

Supplementary Material

Cook, D. C., and Danforth, M. E. (2022). Meningiomas in ancient human populations. *Cancers*, *14*(4), 1058.

Cucu, A. I., Costea, C. F., Perciaccante, A., Carauleanu, A., Turliuc, S., Costachescu, B., and Turliuc, M. D. (2019). The history of Arachne through historic descriptions of meningiomas with hyperostosis: from prehistory to the present. *World Neurosurgery*, *128*, 37-46.

Gorlick, R., and Khanna, C. (2010). Osteosarcoma. *Journal of bone and mineral research*, *25*(4), 683-691.

Huchet, J. B., Deverly, D., Gutierrez, B., and Chauchat, C. (2011). Taphonomic evidence of a human skeleton gnawed by termites in a Moche-civilisation grave at Huaca de la Luna, Peru. *International Journal of Osteoarchaeology*, *21*(1), 92-102.

Lieverse, A. R., Temple, D. H., and Bazaliiskii, V. I. (2014). Paleopathological description and diagnosis of metastatic carcinoma in an Early Bronze Age (4588+ 34 Cal. BP) forager from the Cis-Baikal region of Eastern Siberia. *PLoS One*, *9*(12), e113919.

Marks, M. K., and Hamilton, M. D. (2007). Metastatic carcinoma: palaeopathology and differential diagnosis. *International Journal of Osteoarchaeology*, *17*(3), 217-234.

Marques, C. (2019). Tumors of bone. In *Ortner's identification of pathological conditions in human skeletal remains* (pp. 639-717). Academic Press.

Nikita, E. (2016). *Osteoarchaeology: A guide to the macroscopic study of human skeletal remains*. Academic Press.

O'Connell, L. (2004). Guidance on recording age at death in adults. *Brickley M, McKinley J. Guidelines to the Standards for Recording Human Remains. Department of Archaeology, University of Southampton and Institute of Field Archaeologists, University of Reading*

Ortner, D. J. (2003). Tumors and Tumor-like Lesions of Bone. In *Identification of pathological Conditions in Human Skeletal Remains*, Academic press, 503-544.

Ortner, D. J., Ponce, P., Ogden, A., and Buckberry, J. (2012). Multicentric osteosarcoma associated with DISH, in a 19th century burial from England. *International Journal of Osteoarchaeology*, *22*(2), 245-252.

Pechenkina, K., Fan, W., and Luo, X. (2019). What's that big thing on your head? Diagnosis of a large frontoparietal lesion on an Eastern Zhou skull from Henan, China. *International Journal of Paleopathology*, *26*, 84-92.

Quinn, K. (2017). *A bioarchaeological study of the impact of mobility on the transmission of tuberculosis in Roman Britain* (Doctoral dissertation, Durham University).

Rubini, M., Gozzi, A., Libianchi, N., Dellu, E., Spano, F., Di Biasi, C., and Zaio, P. (2022). Metastatic cancer and endentulism: Exploring comorbidity to assist with differential diagnosis in a case from Vico nel Lazio (Fr, Italy), 13th-15th century CE. *International Journal of Paleopathology*, *38*, 1-12.

Schultz, M., Parzinger, H., Posdnjakov, D. V., Chikisheva, T. A., and Schmidt-Schultz, T. H. (2007). Oldest known case of metastasizing prostate carcinoma diagnosed in the skeleton of a 2,700-year-old Scythian king from Arzhan (Siberia, Russia). *International Journal of Cancer*, *121*(12), 2591-2595.

Shang, H., and Trinkaus, E. (2008). An ectocranial lesion on the Middle Pleistocene human cranium from Hulu Cave, Nanjing, China. *American Journal of Physical Anthropology: The Official Publication of the American Association of Physical Anthropologists*, *135*(4), 431-437.

Strouhal, E. (1991). Myeloma multiplex versus osteolytic metastatic carcinoma: differential diagnosis in dry bones. *International Journal of Osteoarchaeology*, *1*(3-4), 219-224.

Strouhal, E., Vyhnanek, L., Horáčková, L., Benesova, L. and Nemecková,, A. (1997). A case of osteosarcoma in a late medieval–early modern skull from Kyjov (Czech Republic). *International Journal of Osteoarchaeology*, *7*(1), 82-90.

Verano, J. W. (2016). Differential diagnosis: trepanation. *International Journal of Paleopathology*, 14, 1-9.

White T., and Folkens, P. 2005. *The Human Bone Manual*. New York, Academic Press.

White, T. D., Black, M. T., and Folkens, P. A. (2011). *Human osteology*. Academic press.

Wu, X. J., Schepartz, L. A., Liu, W., and Trinkaus, E. (2011). Antemortem trauma and survival in the late Middle Pleistocene human cranium from Maba, South China. *Proceedings of the National Academy of Sciences*, *108*(49), 19558-19562.
